# New Insights into Xenotransplantation for Cartilage Repair: Porcine Multi-Genetically Modified Chondrocytes as a Promising Cell Source

**DOI:** 10.3390/cells10082152

**Published:** 2021-08-20

**Authors:** Hanna Tritschler, Konrad Fischer, Jochen Seissler, Jörg Fiedler, Rebecca Halbgebauer, Markus Huber-Lang, Angelika Schnieke, Rolf E. Brenner

**Affiliations:** 1 Division for Biochemistry of Joint and Connective Tissue Diseases, Department of Orthopedics, University of Ulm, 89081 Ulm, Germany; hanna.tritschler@uni-ulm.de (H.T.); joerg.fiedler@uni-ulm.de (J.F.); 2 Chair of Livestock Biotechnology, School of Life Sciences Weihenstephan, Technische Universität München, 85354 Freising, Germany; konrad.fischer@wzw.tum.de (K.F.); angelika.schnieke@wzw.tum.de (A.S.); 3 Medizinische Klinik und Poliklinik IV, Diabetes Zentrum—Campus Innenstadt, Klinikum der Ludwig-Maximilians-Universität, 80336 München, Germany; jochen.seissler@med.uni-muenchen.de; 4 Institute of Clinical and Experimental Trauma Immunology, University Hospital Ulm, 89081 Ulm, Germany; rebecca.halbgebauer@uniklinik-ulm.de (R.H.); markus.huber-lang@uniklinik-ulm.de (M.H.-L.)

**Keywords:** porcine chondrocytes, xenotransplantation, complement, α-1,3-Gal-epitope, Neu5Gc-epitope, CD46, CD55, CD59, TNFAIP3, heme oxygenase-1

## Abstract

Transplantation of xenogenic porcine chondrocytes could represent a future strategy for the treatment of human articular cartilage defects. Major obstacles are humoral and cellular rejection processes triggered by xenogenic epitopes like α-1,3-Gal and Neu5Gc. Besides knockout (KO) of genes responsible for the biosynthesis of respective epitopes (GGTA1 and CMAH), transgenic expression of human complement inhibitors and anti-apoptotic as well as anti-inflammatory factors (CD46, CD55, CD59, TNFAIP3 and HMOX1) could synergistically prevent hyperacute xenograft rejection. Therefore, chondrocytes from different strains of single- or multi-genetically modified pigs were characterized concerning their protection from xenogeneic complement activation. Articular chondrocytes were isolated from the knee joints of WT, GalTKO, GalT/CMAH-KO, human CD59/CD55//CD46/TNFAIP3/HMOX1-transgenic (TG), GalTKO/TG and GalT/CMAHKO/TG pigs. The tissue-specific effectiveness of the genetic modifications was tested on gene, protein and epitope expression level or by functional assays. After exposure to 20% and 40% normal human serum (NHS), deposition of C3b/iC3b/C3c and formation of the terminal complement complex (TCC, C5b-9) was quantified by specific cell ELISAs, and generation of the anaphylatoxin C5a by ELISA. Chondrocyte lysis was analyzed by Trypan Blue Exclusion Assay. In all respective KO variants, the absence of α -1,3-Gal and Neu5Gc epitope was verified by FACS analysis. In chondrocytes derived from TG animals, expression of CD55 and CD59 could be confirmed on gene and protein level, TNFAIP3 on gene expression level as well as by functional assays and CD46 only on gene expression level whereas transgenic HMOX1 expression was not evident. Complement activation in the presence of NHS indicated mainly effective although incomplete protection against C3b/iC3b/C3c deposition, C5a-generation and C5b-9 formation being lowest in single GalTKO. Chondrocyte viability under exposure to NHS was significantly improved even by single GalTKO and completely preserved by all other variants including TG chondrocytes without KO of xenoepitopes.

## 1. Introduction

The treatment of articular cartilage injuries remains a major clinical and experimental challenge. Cell-based therapeutic approaches are still largely limited by the availability of sufficient chondrogenic cells with a phenotypically stable differentiated phenotype after transplantation. The use of autologous chondrocytes from non-load-bearing articular regions is associated with a two-step surgical procedure and the risk of donor-site morbidity [1]. Furthermore, the extensive proliferation of chondrocytes in vitro is associated with cellular dedifferentiation which could impair subsequent generation of functional hyaline-like cartilage. Therefore, the use of autologous chondrocytes from the nasal septum has been suggested as an interesting alternative [2]. This strategy, however, based on chondrocytes of different germ-layer origin, does not eliminate the need for a two-step process and the potential risk of donor site morbidity in the nose [3]. Finally, multipotent mesenchymal stem/progenitor cells from different tissues including bone marrow have been studied as a potential autologous or even allogenic cell source for cartilage repair. Despite the considerable proliferative capacity and chondrogenic differentiation potential, phenotypic instability and the subsequent terminal differentiation remains an unsolved problem [4].

In recent decades, xenotransplantation approaches have been developed and continuously optimized for a wide range of organ/cell types in order to solve the persistent shortage of available cell/tissue transplants [5]. From those studies, it is well known that human natural antibodies, reactive with the α-1,3-Gal epitope, initiate hyperacute rejection which is mainly driven by activation of the hosts’ classical complement pathway. This first stage of rejection can be significantly reduced by inactivation of the GGTA1 gene, encoding α-1,3-galactosyl transferase that synthesizes the α-1,3-Gal epitopes on the cell surface [6]. However, other genes such as the cytidine monophospho-N-acetylneuraminic acid hydroxylase (CMAH), creating the xenoreactive Neu5Gc non-Gal epitope, also have to be considered [7,8,9]. Additional incorporation of human complement regulatory proteins (hCregs) further enhances the prevention of hyperacute rejection [10]. In general, Cregs are necessary to balance complement activation and protect host cells against autologous complement attack. They are present in a soluble or membrane-bound manner. Membrane cofactor protein (MCP or CD46), decay-accelerating factor (DAF or CD55) and the terminal complement complex (TCC) inhibitor CD59 act on different stages of the complement cascade. In early complement activation, CD55 impedes the assembly of C3 convertase (C3bBb or C4b2a) by its cleavage into C3b and Bb or C4b and C2a [11,12]. Likewise, CD46 has an effect on the same complement stage by binding to C3b or C4b [13]. Initiation of the terminal pathway starts with the cleavage of C5 into the anaphylatoxin C5a and C5b, the latter resulting in the self-assembly of the complement components C5b, C6, C7, C8 and C9, which form the TCC. This leads to pore formation in the cell membrane and finally cell lysis or pro-inflammatory reaction when existent in sublytic amounts. The Creg CD59 (also known as Protectin) prevents C9 polymerization into a C5b-9 complex [14]. Blockade of xenogenic antibody-triggered complement activation prevents hyperacute rejection, but fails to inhibit acute vascular rejection which is not a major concern in cartilage repair. Nevertheless, candidates primarily selected to control acute vascular rejection like hemoxygenase1 (HMOX1) and zinc-finger protein TNFAIP3 [15] may also have positive effects in non-vascularized tissues because of their cytoprotective properties. HMOX1 is an inducible, microsomal enzyme, which catalyzes the cleavage of cytotoxic heme into equimolar CO, free iron and biliverdin via oxidation [16,17]. A variety of physical and chemical stress stimuli, like cytokines (e.g., TNF, IL1, IL6), nitric oxide, and prostaglandins can initiate the actions of HMOX1 [17]. The resulting reaction products finally act via different cellular signaling pathways to develop anti-apoptotic, -inflammatory and -oxidative effects [18,19]. The cytoplasmic TNFAIP3 (known as A20) acts in a different cytoprotective manner than HMOX1. It is an important regulator of inflammation and immune homeostasis because of its feedback inhibitory activity on the transcription factor NF-kB. A wide range of extracellular stimuli like growth factors, cytokines (TNF, IL1) and TLRs and their downstream signaling effectors activate NF-kB and subsequent expression of TNFAIP3 [20,21]. In the context of osteoarthritis, NF-κB is known to be involved in the downregulation of proteoglycan and collagen type II synthesis [22,23] and stimulation of matrix-degrading enzyme expression, including aggrecanases (ADAMTS4, ADAMTS5) and matrix metalloproteinases [24], like MMP13 [23,25]. Furthermore, it initiates the expression of inducible nitric oxide synthase (iNOS) and cyclooxygenase 2 (COX2) responsible for NO and PGE2 synthesis [25].

So far, only a few studies have addressed the hypothesis that genetically modified porcine chondrocytes could represent a promising future cell source for human cartilage repair [26,27,28], mainly concentrating on the suppression of α-1,3-Gal-mediated antibody responses by transgenic expression of α1,2-fucosyltransferase in pigs, in vitro transfection of porcine chondrocytes and inhibition of specific molecules to identify potential targets for chondrocyte xenoprotection.

Therefore, the present study aimed at the characterization of multi-genetically modified porcine chondrocytes (1. variant: GGTA1 inactivation; 2. variant: GGTA1 and CMAH inactivation; 3. variant: incorporation of five human genes (CD59/CD55/CD46/TNFAIP3/HMOX1); 4. variant: combination of group 1 and 3; 5. variant: combination of group 2 and 3) with respect to histomorphological properties of articular cartilage tissue as well as α-1,3-Gal, Neu5Gc and transgene expression of isolated chondrocytes. Furthermore, the protective effects of GGTA1 without and with CMAH inactivation and five-fold human transgene expression, alone or in combination, on complement-activation and complement-dependent cytolysis were investigated. The comparative study of five variants of genetically modified pigs allowed us to thoroughly assess their relative impact on complement-mediated xenorejection processes in vitro.

## 2. Material and Methods

### 2.1. Generation of Multi-Transgenic Pigs

As previously reported, multi-genetically modified pigs were generated by human transgene addition or gene inactivation of CMAH and GGTA1 (CRISPR/Cas9 technology) followed by somatic nuclear transfer [15]. Animal experiments were approved by the Government of Upper Bavaria (permit number 55.2-1-54-2532-6-13) and performed according to the German Animal Welfare Act and European Union Normative for Care and Use of Experimental Animals. In the present study, we used the articular cartilage of young pigs from shortly after birth up to 2 years. Table 1 listed the used abbreviations for the individual genetic variants:

### 2.2. Isolation of Porcine Chondrocytes and Cell Culture

Cartilage of porcine femoral condyles of WT, TG, GalTKO, GalTKO/TG, GalT/CMAHKO and GalT/CMAHKO/TG animals were minced and digested with pronase (9 U/mL; 45 min; MERCK, Darmstadt, Germany) and collagenase (U/mL; about 8 h; MERCK, Darmstadt, Germany) at 37 °C. The cells were expanded in FBS-containing medium consisting DMEM/Ham’s F12 (1:1; Thermo Fisher Scientific, Germering, Germany, and PAN Biotech, Aidenbach, Germany), 10% heat-inactivated fetal bovine serum, 0.5% penicillin/streptomycin (PAA Laboratories, Pasching, Germany), 0.5% l-glutamine (PAA Laboratories, Pasching, Germany) and 10 µg/mL 2-phospho-l-ascorbic acid trisodium salt (MERCK, Darmstadt, Germany) at 37 °C, 5% CO_2_ and 95% humidity [29].

### 2.3. AlamarBlue^®^ Cell Proliferation Assay

For the quantification of chondrocyte proliferation within one week of cultivation, an AlamarBlue^®^ (Bio-Rad, Feldkirchen, Germany) assay was performed as previously described [30,31]. After 24 h adherence on culture, plastic culture medium was removed and AlamarBlue^®^ solution (5% in cultivation medium) was added. After 3 h incubation at 37 °C, fluorescence was measured at 555 nm excitation and 590 nm emission by a microplate reader (Infinite M200, Tecan Deutschland, Crailsheim, Germany). This time point was defined as t0 and the AlamarBlue^®^ solution was replaced by the cultivation medium. After a further 1, 4 and 7 days of cultivation, the analysis was repeated and proliferation activity was calculated in relation to t0.

### 2.4. Immunohistochemistry (IHC) and Histology

Sections of EDTA-decalcificated and paraffin-embedded femoral condyles were dewaxed and rehydrated prior to staining of proteoglycans with Safranin O [32]. For collagen type II IHC endogenous peroxidases were blocked by 3% H_2_O_2_ and pre-digested with pepsin (1 mg/mL in 0.5 M acetic acid) for antigen-retrieval. Further staining was performed using Agilent LSAB2 System HRP kit and antibody against collagen type II (II-II6B3, Developmental Studies Hybridoma Bank).

To visualize C5b-9 formation after serum exposure, 0.2 × 10^5^ cells were seeded on culture slides and treated with 20% pooled normal human serum (NHS, Innovative Research, Novi, MI, USA) in FBS-free medium. FBS-free medium alone served as control. After 1 h NHS exposure, cells were fixed with Lillie’s formalin and peroxidase blocking was performed. Cells were incubated with anti-human SC5b-9 (Quidel, San Diego, CA, USA; 1:5000) overnight at 4 °C. Antibody binding was visualized by Agilent LSAB2 System HRP and Meyer’s hemalum solution was used for counterstaining.

### 2.5. Immunofluorescence Staining and Flow Cytometric-Based Expression Analysis

Chondrocytes were seeded on culture slides and adherent cells were incubated with GS IB4-FITC (Biozol, Eching, Germany; 1:50 in stimulation medium) for 30 min. Unbound GS IB4-FITC was removed by washing with PBS and subsequent DAPI (0.25 µg/mL) cell nuclei staining was performed (15 min). After mounting with fluorescent mounting medium (Agilent Technologies, Waldbronn, Germany), cells were analyzed by fluorescence microscopy (Zeiss Axioskop 2 mot plus; Zeiss, Oberkochen, Germany). For flow cytometric analysis, chondrocytes were detached by trypsin/EDTA treatment (concentration 0.05%/0.02%) and a minimum of 1 × 10^5^ cells were incubated with GS IB4-FITC for 30 min or immuno-cytologically stained for 20 min in dark with one of the following antibodies or corresponding isotype controls: mouse anti-human CD46 FITC (#315304, 1:5), anti-human CD55 APC (#311312, 1:20), anti-human CD59 PE (#304708, 1:50), IgG1 FITC (#400108, 1:5), IgG1 APC (#400120, 1:20) and IgG2a PE (#400212, 1:50) (all obtained from BioLegend, San Diego, CA, USA). Data were acquired using a FACSCanto II (BD Biosciences, Heidelberg, Germany). In case of Neu5Gc detection, porcine chondrocytes were cultured for at least 2 weeks in medium consisting DMEM/Ham’s F12 (1:1), 5% heat-inactivated normal human serum (Innovative Research, Novi, MI, USA), 0.5% penicillin/streptomycin, 0.5% L-glutamine and 10 µg/mL 2-phospho-l-ascorbic acid trisodium salt at 37 °C, 5% CO_2_ and 95% humidity to remove Neu5Gc cross-contamination [9]. Cells were detached with EDTA (5 mM, pH 7.2) and incubated with chicken anti-Neu5Gc IgY or IgY isotype (Poly21469, BioLegend) for 1 h at 4 °C. A subsequent washing step with PBS was performed before staining with donkey anti-chicken IgY FITC conjugated antibody (Jackson ImmunoResearch, West Grove, PA, USA) for 1 h in the dark at 4 °C. Neu5Gc-positive cells were measured by FACS Calibur (BD BioSciences, Heidelberg, Germany). Flow cytometric measurements were analyzed using FlowJo^TM^ Software for macOS, version 10.2 (FlowJo LLC., Ashland, OR, USA).

### 2.6. Expression of Transgenes and Cartilage ECM Associated Genes

RNA was isolated as previously described [29] and cDNA was synthesized using SuperScript II kit (Thermo Fisher Scientific, Germering, Germany) according to the manufacturer’s instructions. GoTaq^®^ DNA-Polymerase (Promega, Waldorf, Germany) was utilized for semi-quantitative RT-PCR. Expression of transgenes CD46, CD55, CD59, HMOX1 and TNFAIP3 were determined whereas porcine GAPDH was used as endogenous control. Likewise, porcine ECM-associated genes COL2A1 and ACAN were investigated. For each primer, a final concentration of 2.4 µM and 1 µL cDNA was used for the reaction (Oligonucleotide sequences in Table S1).

### 2.7. Detection of TNFAIP3 Activity

TNFAIP3 activity is indirectly detectable by both TNF exposure following measurement of NO production or determination of Caspase-8 activity after TNF and cycloheximide (CHX; Merck, Darmstadt, Germany) treatment [15]. For detection of NO production, chondrocytes were stimulated with 5 ng/mL TNF (Thermo Fischer Scientific, Germering, Germany) for 24 h, supernatant fluids were collected and cells were digested with proteinase K for DNA content calculation by Hoechst staining (Fluka Chemie GmbH, Seelze, Germany). The stable metabolite nitrite was analyzed in supernatants by Griess Assay (Promega, Waldorf, Germany) according to the manufacturer’s instructions. Apoptosis was induced by 10 ng/mL TNF and 10 µg/mL cycloheximide (Merck, Darmstadt, Germany). After 5 h induction, Caspase Glo-8 (Promega, Waldorf, Germany) was performed by adding 70 µL of Caspase substrate followed by 1.5 h incubation before measuring luminescence by using multimode microplate reader Infinite M200 Pro (Tecan, Crailsheim, Germany).

### 2.8. Anaphylatoxin Generation

To analyze anaphylatoxin C5a generation by complement activation, 0.7 × 10^5^ chondrocytes were exposed to 40% NHS for 30 min at 37 °C in a reaction tube. Cell-free NHS was used as control. A subsequent centrifugation step was performed at 200× *g* (Biofuge, Thermo Fisher Scientific, Germering, Germany) for 10 min, supernatants were collected and C5a concentrations measured using a human C5a ELISA kit (Thermo Fisher Scientific, Germering, Germany) according to the manufacturer’s instruction.

### 2.9. Trypan Blue Exclusion Assay

To investigate complement-mediated lysis, the protocol of Wang et al. [33] was adapted. Overall, 2 × 10^5^ chondrocytes per tube were incubated in various dilutions of NHS w/wo heat-inactivation in a total volume of 100 µL for 1 h at 37 °C. Afterwards, the cell suspension was mixed with 0.4% trypan blue and living as well as dead cells were counted in a hemocytometer (Neubauer Improved, Brand GmbH, Wertheim, Germany) for calculating survival rate.

### 2.10. C5b-9 and Activated C3b Specific Cell ELISA

Chondrocytes were seeded in duplicates on 96-well cell culture plates (0.7 × 10^5^ cells/well) and after 24 h adherence, cells were rinsed with PBS and exposed to FBS-free medium (DMEM/Ham’s F12, 0.5% l-glutamine, 0.5% penicillin/streptomycin) supplemented with normal human serum (NHS) or heat-inactivated (HI) NHS for 1 h, respectively. The cell ELISA was performed as previously described [34]. Briefly, after NHS treatment (20% and 40%), cells were fixed and prior to primary antibody incubation with rabbit polyclonal C5b-9 (abcam, Cambridge, UK), in case of C5b-9 deposition, and with mouse anti-human activated C3 (1:500, clone bH6, HycultBiotech, Wayne, PA, USA) in case of C3b/iC3b/C3c deposition, cells were blocked with 5% bovine serum albumin (Merck, Darmstadt, Germany). As secondary antibodies, an HRP-conjugated anti-rabbit IgG (Merck, Darmstadt, Germany) or HRP-conjugated goat anti-mouse IgG2a (1:2000, Thermo Fisher Scientific, Germering, Germany) antibody was used. To visualize C5b-9 or C3b deposition 3,3′,5,5′-tetramethylbenzidine (TMB; Merck, Darmstadt, Germany) was added and absorbance at 450 nm was measured. For normalization, DNA content was determined by subsequent Hoechst staining.

### 2.11. Statistics

Values are given as scatter dot plots with mean ± standard deviation or boxplots (median; whiskers: min to max). Experiments were statistically analyzed by using GraphPad Prism (version 9.0.0 for Windows, GraphPad Software, San Diego, CA, USA). Differences were always considered to be significant when *p* < 0.05. Depending on the experiment setup, “one-way ANOVA” or “two-way ANOVA” with Dunnett’s multiple comparisons test was performed.

## 3. Results

### 3.1. Genetically Modified Chondrocytes Show No Obvious Biological Disadvantage

**Figure 1:**
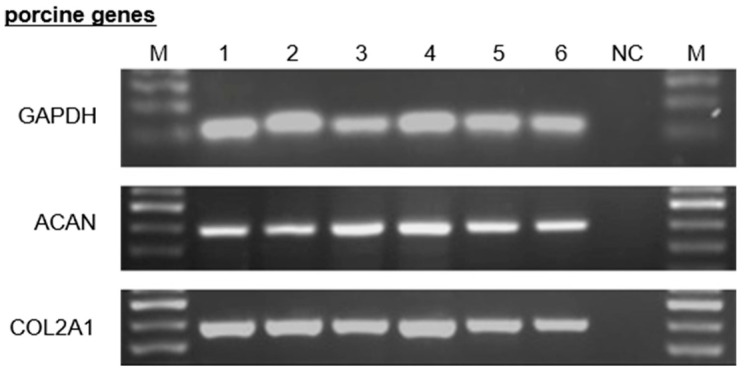
Expression of porcine ECM related genes. Representative RT-PCR of WT and genetically modified porcine chondrocytes regarding porcine COL2A1 and ACAN, whereas porcine GAPDH served as control. The corresponding samples were loaded for electrophoresis in the following order: lane M: bp marker; lane 1: WT; lane 2: TG; lane 3: GalTKO; lane 4: GalTKO/TG; lane 5: GalT/CMAHKO; lane 6: GalT/CMAHKO/TG; NC: negative control.

To ensure comparable cartilage quality of WT, TG, GalTKO, GalTKO/TG, GalT/CMAHKO and GalT/CMAHKO/TG, femoral condyles were macroscopically evaluated followed by exemplarily histological/immunohistochemical assessment. Cartilage appearance of all variants exhibited a smooth, slippery and continuous surface lacking fibrillation, fissures or cracks. In Safranin O and collagen type II staining cell density and distribution were similar in genetically modified (GalTKO, TG, GalTKO/TG, GalT/CMAHKO and GalT/CMAHKO/TG) compared to WT cartilage. No cell clustering, hypo- or hypercellularity was visible. Likewise, glycosaminoglycan and collagen type II staining intensities and distribution revealed no differences (Figure S1). Semi-quantitative RT-PCR analysis confirmed comparable mRNA expression of COL2A1 and ACAN in all tested variants with comparable GAPDH levels (Figure 1). Within one week of cultivation, cell growth increased for all cell types. The proliferation of genetically modified chondrocytes was at all timepoints similar to WT chondrocytes and there was no significant difference between them. Nevertheless, mean values of variant TG were to some extent lower than the other ones (respective *p*-values were *p* = 0.999 (1d); *p* = 0.782 (4d); *p* = 0.258 (7d) (Figure S2).

### 3.2. Genetically Modified Chondrocytes Show Strong Expression of CD55/CD59/TNFAIP3 and Absence of Xenoantigens α-1,3-Gal and Neu5Gc

**Figure 2:**
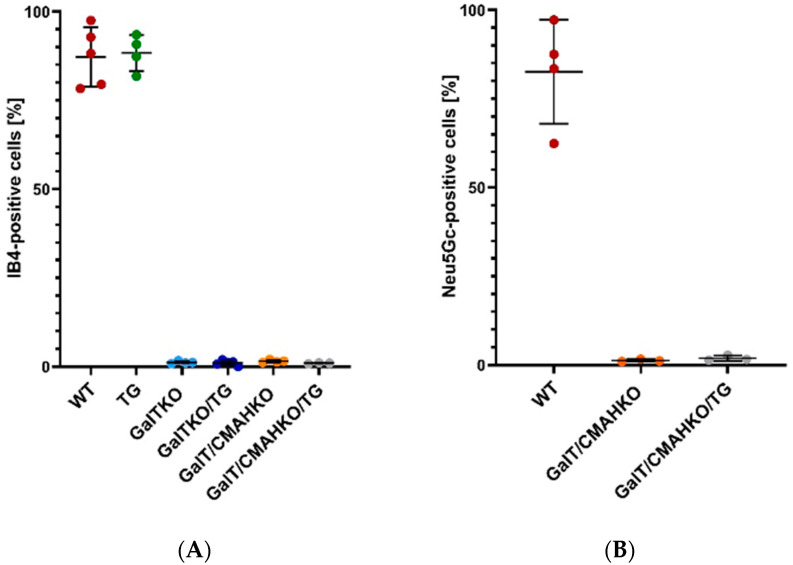
Phenotypical analysis of GGTA1 and CMAH inactivation. Results of surface presence of xenoantigens α-1,3-Gal (**A**) and Neu5Gc (**B**) on WT and genetically modified porcine chondrocytes by flow cytometry. Data values are given relative to total cell number, *n* ≥ 3.

To examine the presence of the α-1,3-Gal epitope, chondrocytes were stained by GS Isolectin B4 (IB4) for flow cytometry as well as fluorescence microscopy. The quantitative comparison between the various genetically modified chondrocytes and the WT confirmed the lack of α-1,3-Gal epitopes in GGTA1 knockout chondrocytes (WT: 87%; TG: 88%; GalTKO: 1%; GalTKO/TG: 1%; GalT/CMAHKO: 1.5%; GalT/CMAHKO/TG: 1%) (Figure 2A). These findings are in accordance with the IB4 fluorescence staining (Figure S3). Functional knockout of CMAH could also be confirmed. In total, 83% of WT chondrocytes were Neu5Gc-positive and the respective detection level was near to zero in CMAH knockout variants (GalT/CMAHKO: 1.3% and GalT/CMAHKO/TG: 1.9%) (Figure 2B). Representative histograms are shown in Figure S4.

**Figure 3:**
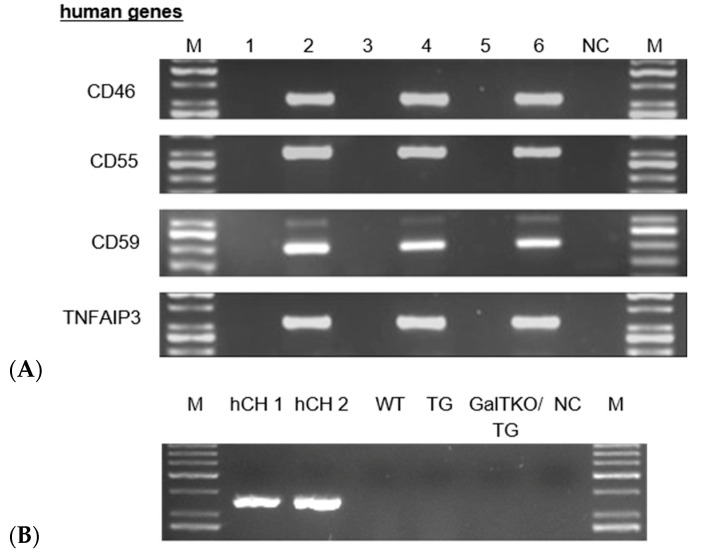
Expression of human transgenes in porcine chondrocytes. (**A**) Representative RT-PCR of WT and genetically modified porcine chondrocytes regarding human CD46, CD55, CD59, TNFAIP3; porcine GAPDH served as control (shown in Figure 1). The corresponding samples were loaded for electrophoresis in the following order: lane M: bp marker; lane 1: WT; lane 2: TG; lane 3: GalTKO; lane 4: GalTKO/TG; lane 5: GalT/CMAHKO; lane 6: GalT/CMAHKO/TG; NC: negative control (**B**) HMOX1 gene expression in human (hCH) and porcine chondrocytes (WT, TG and GalTKO/TG).

**Figure 4:**
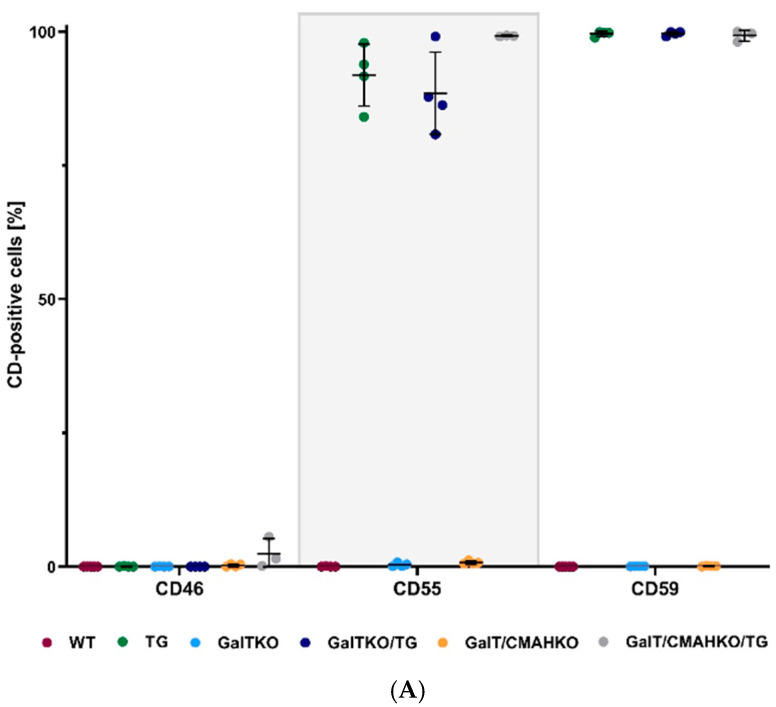

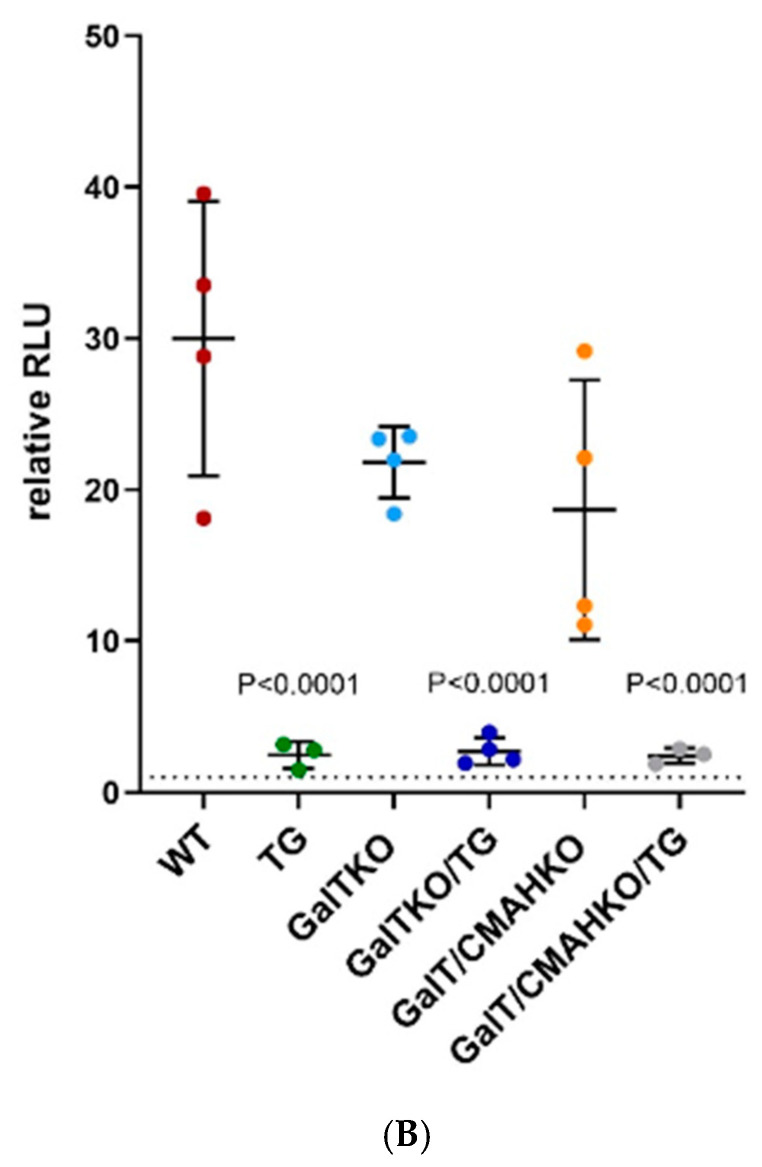
Phenotypical analysis of transgenes in porcine chondrocytes. Results of surface presence of human transgenes CD46, CD55, CD59; (**A**) Human TNFAIP3 expression was indirectly detected by caspase 8 activity (**B**). Therefore, porcine chondrocytes were exposed to TNF (10 ng/mL) and cycloheximide (10 µg/mL) to induce apoptosis. Caspase 8 activity was detected by Caspase Glo-8 assay. Data values are given relative total cell number (**A**) or to not TNF/cycloheximide-stimulated cells (**B**, dotted line). Statistical analysis was performed by one-way ANOVA with Dunnett’s post hoc test and significant differences compared to WT are labeled with the corresponding *p*-values, *n* ≥ 3.

Transgene expression of human TNFAIP3, CD59, CD55 and CD46 could be demonstrated in TG, GalTKO/TG and GalT/CMAHKO/TG chondrocytes by RT-PCR, with the exception of HMOX1 (Figure 3A,B). CD46 surface expression could not be proven in flow cytometric analysis, while membrane-bound CD59 and CD55 were present on TG chondrocytes (99%; 91%), GalTKO/TG (99%; 88%) and GalT/CMAHKO/TG (99%; 99%) compared to WT (0%; 1%), GalTKO (0%; 0%) and GalT/CMAHKO (0%; 1%), respectively as shown in Figure 4A. Representative histograms are presented in the supplementary part (Figure S4). Using the same experimental approach, CD46 could be detected in over 90% of human osteoarthritic chondrocytes as a positive control (data not shown). On a protein level, A20 could only be detected by indirect methods to assess TNFAIP3 activity since no antibodies were available to distinguish between human and porcine origin. Induction of apoptosis via TNF and CHX resulted in an increased caspase 8 activity in WT, GalTKO and GalT/CMAHKO chondrocytes compared to not TNF/CHX-stimulated cells. In contrast, all transgenic variants (TG, GalTKO/TG and GalT/CMAHKO/TG) exhibited a significant reduction in caspase 8 activity (Figure 4B). Furthermore, production of nitric oxide measured by the stable metabolite nitrite in porcine chondrocytes after TNF stimulation confirmed these results. Nitrite production was significantly decreased in TG, GalT/CMAHKO/TG and by the tendency in GalTKO/TG chondrocytes in relation to the WT. Nitrite amounts in supernatants of GalTKO and GalT/CMAHKO were comparable with WT supernatants (Figure S5).

### 3.3. Knockout of Xenoantigens and/or Expression of hCregs Result in Protection from Complement-Mediated Destruction of Chondrocytes

**Figure 5:**
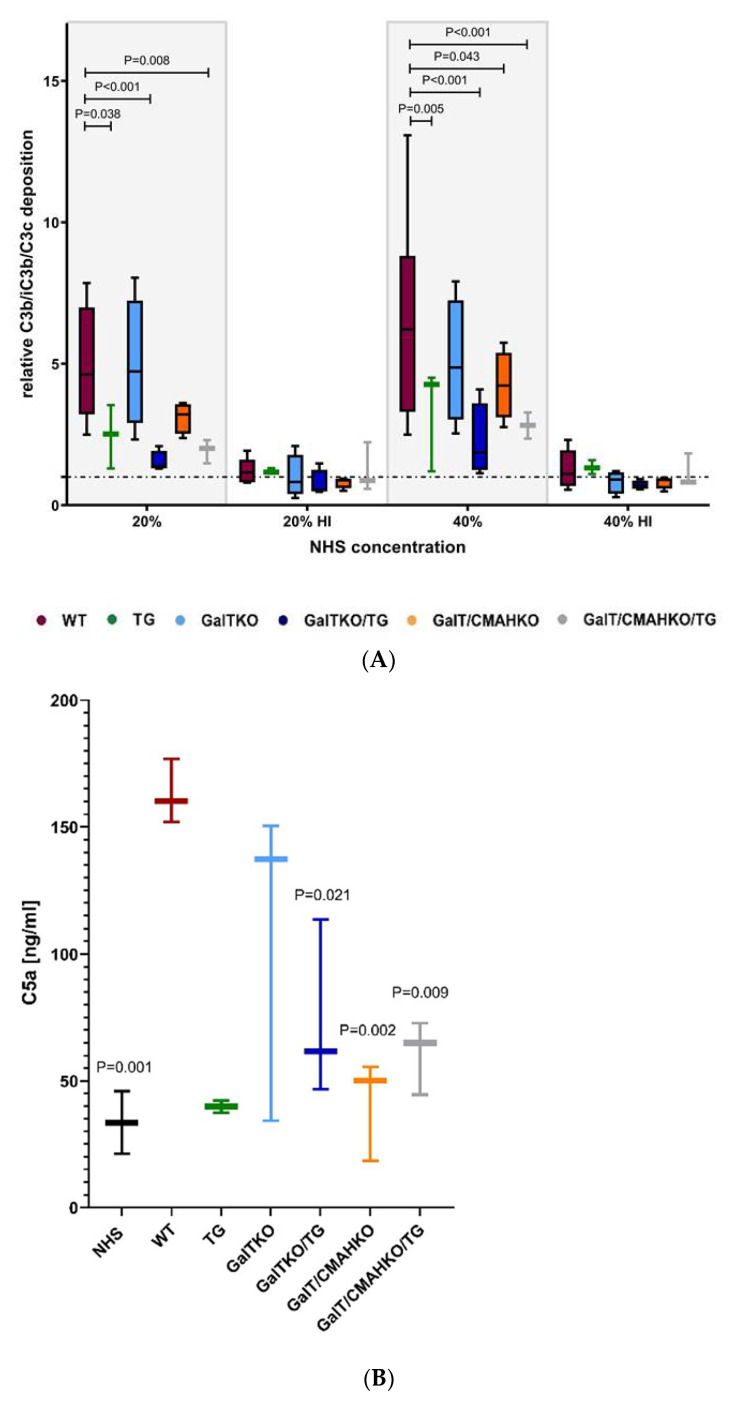
Xenoprotective properties of genetically modified chondrocytes on C3/C5 convertase level. Activated C3 (C3b, iC3b, C3c) deposition on porcine chondrocytes were quantified by specific cell ELISA after exposure the NHS (20% or 40% and heat-inactivated). Results were normalized to DNA content (determined by Hoechst staining) and are given in the diagram as ratio to unstimulated chondrocytes (dotted line) (**A**). Additional, C5a formation was determined in supernatants of NHS (40%) treated chondrocytes and NHS alone served as control (**B**). The variant TG was excluded from the statistical analysis due to low number of donors. Statistical analysis was done by two-way (A) or one-way ANOVA (B) with Dunnett’s multiple comparisons test. *p*-values are given for significant differences compared to WT, *n* = 3–6.

Xenoprotective properties of genetically modified chondrocytes were assessed on various stages of the complement cascade. On C3 level, deposition of activated C3 (C3b, iC3b and C3c) on chondrocytes was determined after NHS exposure. Deposition was detected on any variant, but it was significantly reduced on TG, GalTKO/TG, GalT/CMAHKO and GalT/CMAHKO/TG chondrocytes compared to WT. GGTA1 inactivation resulted in a slightly decreased deposition. Heat-inactivated NHS had no impact on the cells (Figure 5A), indicating complement was responsible for these effects.

Anaphylatoxins are generated in the course of complement activation. Therefore, the impact of genetic modifications was investigated on the C5 level by the anaphylatoxin C5a determination. Significantly more C5a was found in supernatants of NHS-exposed WT chondrocytes (163 ng/mL) than in NHS alone. Transgenic expression of hCregs markedly decreased C5a formation compared to WT. On the contrary, GGTA1 inactivation had only a slight effect (mean value 107 ng/mL), but in combination with hCregs and/or CMAH inactivation there were significant reductions detectable (GalTKO/TG: 74 ng/mL; GalT/CMAHKO: 41 ng/mL; GalT/CMAHKO/TG: 60 ng/mL as mean values) (Figure 5B).

**Figure 6:**
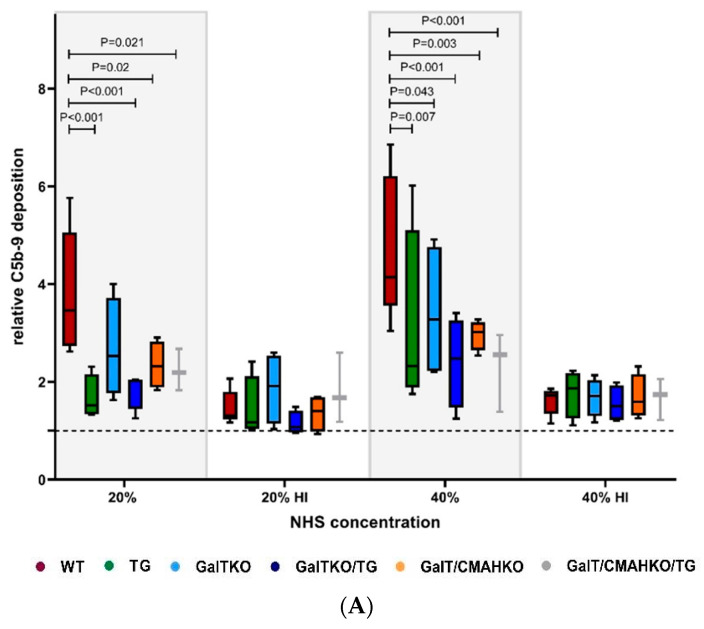

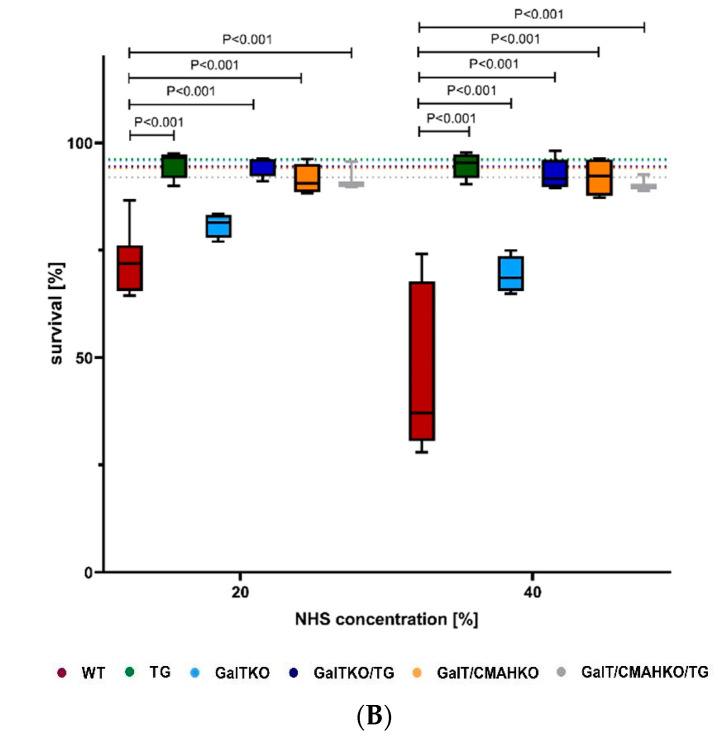
Xenoprotective properties of genetically modified chondrocytes on terminal pathway of complement activation. (**A**) After NHS exposure (20% or 40% and heat-inactivated), C5b-9 deposition on porcine chondrocytes were quantified by specific cell ELISA. DNA content was determined by Hoechst staining and used to normalize C5b-9 deposition. Data values are given as a ratio to unstimulated chondrocytes (dotted line), *n* = 3–5. (**B**) Detection of cytolysis of porcine chondrocytes after NHS exposure (20% or 40%, as well as heat-inactivated). Mean survival rates of the unstimulated variants are indicated as a dotted line in corresponding colors. Statistical analysis was done by two-way ANOVA with Dunnett’s multiple comparisons test. *p*-values are shown in case of significant differences compared to WT, *n* = 3–7.

The effects of GalTKO, CMAHKO and hCreg expression on complement-mediated cytotoxicity were examined by testing the deposition of C5b-9, and sensitivity of WT and genetically modified chondrocytes to NHS in a Trypan Blue Exclusion Assay. Increasing concentrations of NHS resulted in a significantly enhanced formation of C5b-9 (Figure 6A) compared to untreated WT cells and in a dose-dependent reduction in cell viability (Figure 6B). Exposure to 20% NHS revealed cell survival of 72%, whereas 40% NHS caused a further significant increase in cytotoxicity (44%). Heat-inactivation prevented the effects of NHS on WT chondrocytes cell viability (data not shown) and was associated with markedly less C5b-9 deposition (Figure 6A). Protection against complement-mediated lysis was to some extent improved in GalTKO (20% NHS: 81%; 40% NHS: 69%) compared to WT chondrocytes. TG, GalTKO/TG, GalT/CMAHKO and GalT/CMAHKO/TG chondrocytes exhibited strong protective effects and maintained their cell viability (Figure 6B). By tendency, C5b-9 was deposited to a lower extent in the cell ELISA on GalTKO chondrocytes compared to WT with 20% NHS (Figure 6A). Significant reductions were observed for all other genetically modified variants including GalTKO with 40% NHS. These results could be confirmed in immuno-cytological stainings of C5b-9. In these experiments, exposure to 20% NHS caused cell lysis, whereby C5b-9 was only detectable on cell membrane fragments of WT chondrocytes. Likewise, no cell lysis was visible on all transgenic, as well as single and double knockout variants (Figure S6).

## 4. Discussion

Current research in pig-to-human xenotransplantation includes strategies to overcome the barrier of hyperacute rejection by preventing or inhibiting complement activation caused by Gal- and non-Gal-epitopes. Promising approaches to modulate hyperacute rejection involve gene knockout of GGTA1 and Neu5Gc gene and transgenic expression of hCregs.

The present study represents the first analysis of cartilage and chondrocytes from multi-genetically modified pigs which allows the comparison of single GGAT1 knockout, combined GGTA1 and CMAH inactivation, effective transgenic expression of human CD55/CD59/TNFAIP3 and the combination of those approaches with the wild-type situation.

Two further human genes, present in the transgene array (HMOX1 and CD46), were not or not efficiently expressed in cartilage tissue. Absence or weak expression levels of HMOX1 were also reported by Fischer et al. [15]. The genetically modified pigs carry multiple copies of CD55 and CD59 transgenes, resulting in a higher expression than observed in immortalized human mesenchymal stem cells (SCP1 cell line). The CD46 transgene is present as a single copy and its protein expression was about 50% of that detected in these human cells. This difference was reflected in the chondrocytes where CD46 expression could be detected on the gene expression level but the amount of protein was below the level of detection in the current assay.

Relevant findings were the normal macroscopical and histomorphological features of the porcine cartilage irrespective of α-1,3-Gal/Neu5Gc-knockout and/or transgenic expression of CD55/CD59/TNFAIP3. Likewise, genetic modifications had no significant effects on the proliferation capacity of chondrocytes. This is an important point since xenogenic chondrocytes should have the capacity to proliferate and build up a functionally intact and long-term stable cartilage tissue after transplantation. The slightly lower proliferation of TG-chondrocytes could possibly be related to strong complement inhibition since an activating function of sublytic C5b-9 deposition has been reported previously for other cell types [35]. The proliferation capacity of TG-chondrocytes, however, should be high enough to generate sufficient cells and after transplantation cell division has to decline in the course of cartilage tissue formation. In general, porcine articular cartilage resembles the articular cartilage of humans with respect to collagen fiber arrangement and biochemical properties [36,37]. Since our tissue samples were obtained from newborn to 2-year-old pigs, the long-term functionality of the articular cartilage of genetically modified animals remains to be investigated.

Regarding xenoprotective properties, the absence of the α-1,3-Gal-epitope leads to an overall reduction of C3 cleavage products and C5b-9 deposition after exposure to NHS in comparison to wild-type chondrocytes. In parallel, knockout of GGAT1 led to a significant xenoprotection in vitro, but not complete rescue of chondrocyte viability in the presence of NHS. These findings are in good agreement with other published data on GGTA1 gene inactivation [30,38,39,40,41] or expression of human α1,2-fucosyltransferase (namely H-transferase), which reduces α-1,3-Gal epitope generation [42,43,44,45]. This finding can be explained by the fact that other cell surface epitopes besides α-1,3-Gal contribute to complement activation due to human preformed antibodies; examples are glycans modified with N-glycolylneuraminic acid (Neu5Gc) and carbohydrate antigen (SDa) synthesized by porcine β-1,4 N-acetylgalactosaminyltransferase-2 (B4GALNT2). Therefore, studies have already been initiated to additionally delete those genes [46]. In this context we could show that additional CMAH inactivation besides GGAT1-knockout leads to a further decrease in C3 opsonins and C5b-9 deposition and results in the maintenance of chondrocyte viability. The achieved transgenic expression of CD55/CD59/TNFAIP3 completely prevented cytolysis alone or in combination with GGAT1 knockout or in association with CMAH inactivation. Since porcine chondrocyte viability under exposure to human serum could be preserved by efficient transgenic expression of CD55/CD59/TNFAIP3 the parallel expression of HMOX1 does not seem to be necessary for the prevention of cell death of chondrocytes in the acute phase. Nevertheless, its anti-apoptotic, anti-inflammatory and anti-oxidative properties might be of interest in the future for mid- and long-term success after xenotransplantation. In line with our data, Sommaggio et al. demonstrated porcine articular chondrocyte lysis caused by NHS. Blockade of natural anti-Gal antibodies by GAS914, a soluble, polymeric form of a Galalpha(1,3)Gal trisaccharide resulted in less C5b-9 and C3/C4 deposition and reduced IgM/IgG binding [27].

Since lower amounts of C5b-9 deposition in so-called sublytic concentrations induce a pro-inflammatory response and the expression of catabolic enzymes (MMP1, MMP3, MMP13, ADAMTS4, ADAMTS5) [47] an optimal suppression of complement activation should be the final aim, also concerning anaphylatoxin generation which leads to the recruitment and stimulation of immune cells. In our approach, C5a generation was markedly reduced in most genetically modified chondrocytes, but to a lower extent in GalTKO chondrocytes. Similar findings have been reported for H-transferase/CD59 double transgenic, as well as H-transferase transgenic with CD59 and CD55 transduced porcine chondrocytes, which generated lower amounts of key complement activation products (C3a, C5a and Bb) in the presence of NHS [26].

Therefore, also with respect to safety aspects, a combination of both strategies—maximal depletion of problematic cell surface structures and transgenic expression of specific complement regulatory proteins—might represent a viable strategy for future therapies.

Complement activation including TCC-formation has been recognized as a central mechanism of the cartilage trauma response including loss of viable chondrocytes [34] and subsequent cartilage degeneration [47]. Therefore, transgenic expression of hCregs CD55 and CD59 and the anti-apoptotic protein TNFAIP3 might yield an additional benefit after transplantation of chondrocytes in the local environment of a previously injured joint with a cartilage defect. In the osteoarthritic milieu, mechanical stress or pro-inflammatory cytokines, like TNF and IL1B, leads to NF-κB-pathway activation, which causes the expression of catabolic factors like NO, MMPs and ADAMTS proteases inducing cartilage breakdown and progression of osteoarthritis [48]. Transgenic expression of TNFAIP3 may have an additional therapeutic effect as an inhibitor of the NF-κB-pathway. So far, TNFAIP3-mediated NF-κB inhibition in cartilage/chondrocytes has not been directly investigated, but overexpression of ABIN-1, a regulator of TNFAIP3, enabled negative regulation of NF-κB signaling resulting in suppression of apoptosis and maintenance of collagen type II and aggrecan gene expression [49]. In the present study, we could show, that porcine chondrocytes expressing human TNFAIP3 are protected against TNF- and CHX-induced apoptosis. Furthermore, TNF-triggered NF-κB-mediated activation of the iNOS pathway was prevented in human TNFAIP3 transgenic porcine chondrocytes. These results are in line with the previously described decrease of NO release after TNF or IL1 exposure in TNFAIP3 overexpressing meniscus cells [50].

The multi-genetic modifications tested in this study may represent a major step to prevent hyperacute rejection of chondrocytes mainly driven by complement activation. Nevertheless, it has to be kept in mind that further processes of xenogeneic rejection have to be addressed. This includes interactions with human monocytes, T- and NK-cells with respect to adhesion and pro-inflammatory cytokine secretion, apparently triggered by CD86 and VCAM-1 [43,51,52]. In the context of other tissues or cell sources, possible solutions to overcome these obstacles might be the inactivation of porcine SLA class I and II, expression of human CD47 or LEA29Y, a human CTLA4-Ig derivate and inhibitor of T-cell stimulation [46,53,54,55,56,57,58,59]. With the exception of CD47, which was reported to be involved in triggering osteoarthritic processes [60], the other candidates might be promising in the context of cartilage repair in synovial joints. If these immunologic mechanisms could be addressed as effectively as the hyperacute rejection mediated by complement activation, xenogenic chondrocytes might finally help to refute the notion that innovation in cartilage repair is at a standstill [61].

## 5. Conclusions

Chondrocytes from multi-genetically modified pigs might represent a promising cell source for cartilage repair because they are protected from humoral rejection in vitro. While cell viability could be completely preserved with exception of single GalTKO, the residual amount of non-lytic complement activation should be addressed by additional strategies.

## Figures and Tables

**Table 1:** Genetic modifications and abbreviation used for transgenic Sus scrofa.

Abbreviation	Genetic Modification
Wild-type (WT)	-
TG	human CD59/CD55/CD46/TNFAIP3/HMOX1 transgenic
GalTKO	GGTA1^−/−^
GalTKO/TG	GGTA1^−/−^ and human CD59/CD55/CD46/TNFAIP3/HMOX1 transgenic
GalT/CMAHKO	GGTA1^−/−^ and CMAH^−/−^
GalT/CMAHKO/TG	GGTA1^−/−^/CMAH^−/−^ and human CD59/CD55/CD46/TNFAIP3/HMOX1 transgenic

## Supplementary Materials

Supplemental Materials are available online at www.mdpi.com/article/10.3390/cells10082152/s1, Figure S1: Macroscopic and histomorphologic analysis of distal femoral condyles; Figure S2: Proliferation of multi-genetically modified chondrocytes; Figure S3: Distribution of α-1,3-Gal xenoantigen on porcine chondrocytes; Figure S4: Representative histograms of transgene, α-1,3-Gal and Neu5Gc flow cytometric analysis; Figure S5: Indirect human TNFAIP3 verification in genetically modified porcine chondrocytes via TNF treatment; Figure S6: C5b-9 deposition on porcine chondrocytes after NHS treatment; Table S1: Oligonucleotide primers for RT-PCR.
