# Supplementary material for: New Insights into Xenotransplantation for Cartilage Repair: Porcine Multi-Genetically Modified Chondrocytes as a Promising Cell Source
Source: Cells. 2021 Aug 20;10(8):2152. doi: 10.3390/cells10082152 (PMC8394410; doi:10.3390/cells10082152)
Supplement: Supplementary file 1 [file cells-10-02152-s001.zip › cells-1340057-supplementary.pdf]

Supplementary Figure S1

A) Porcine femoral condyles

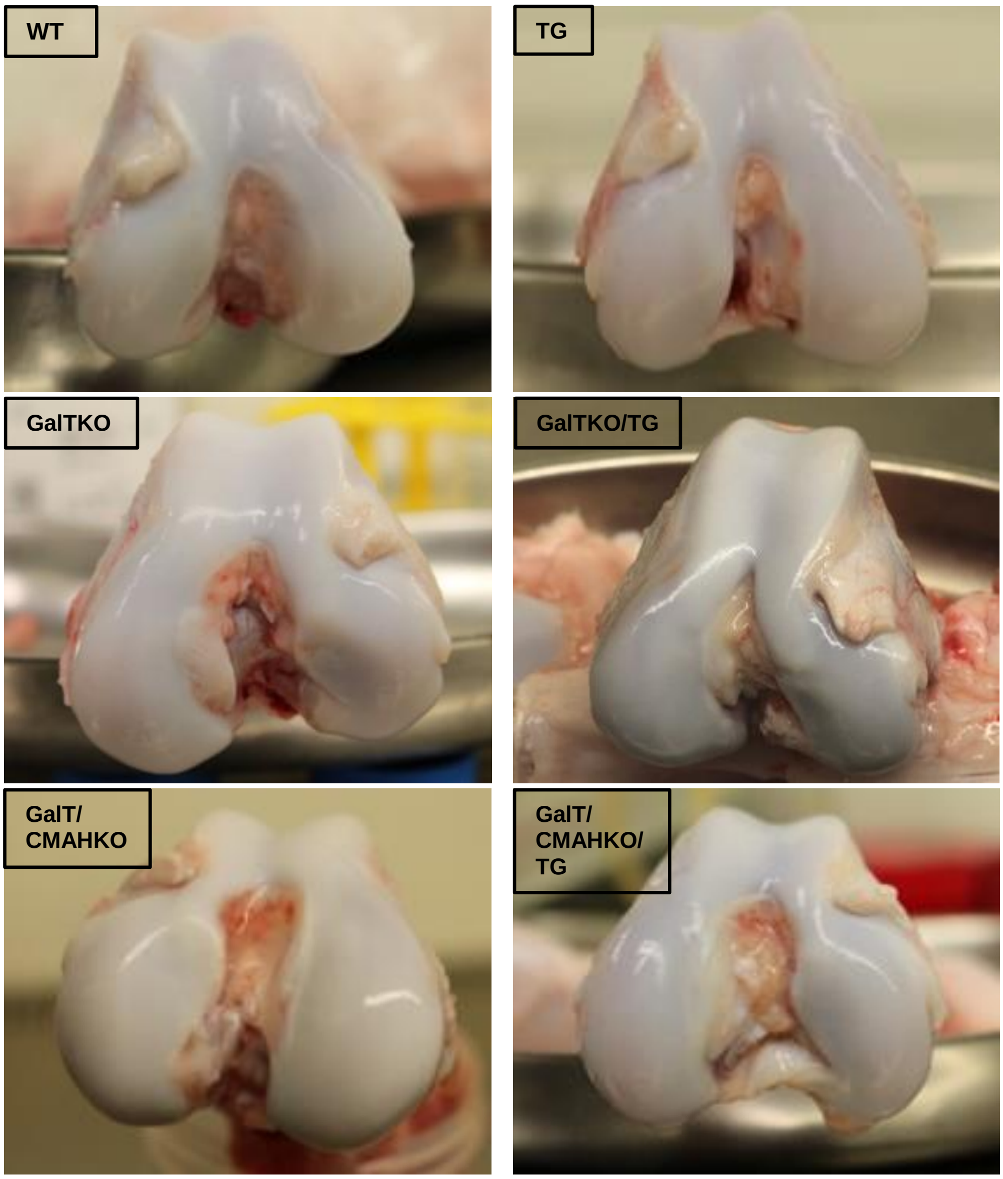

B) Safranin O staining

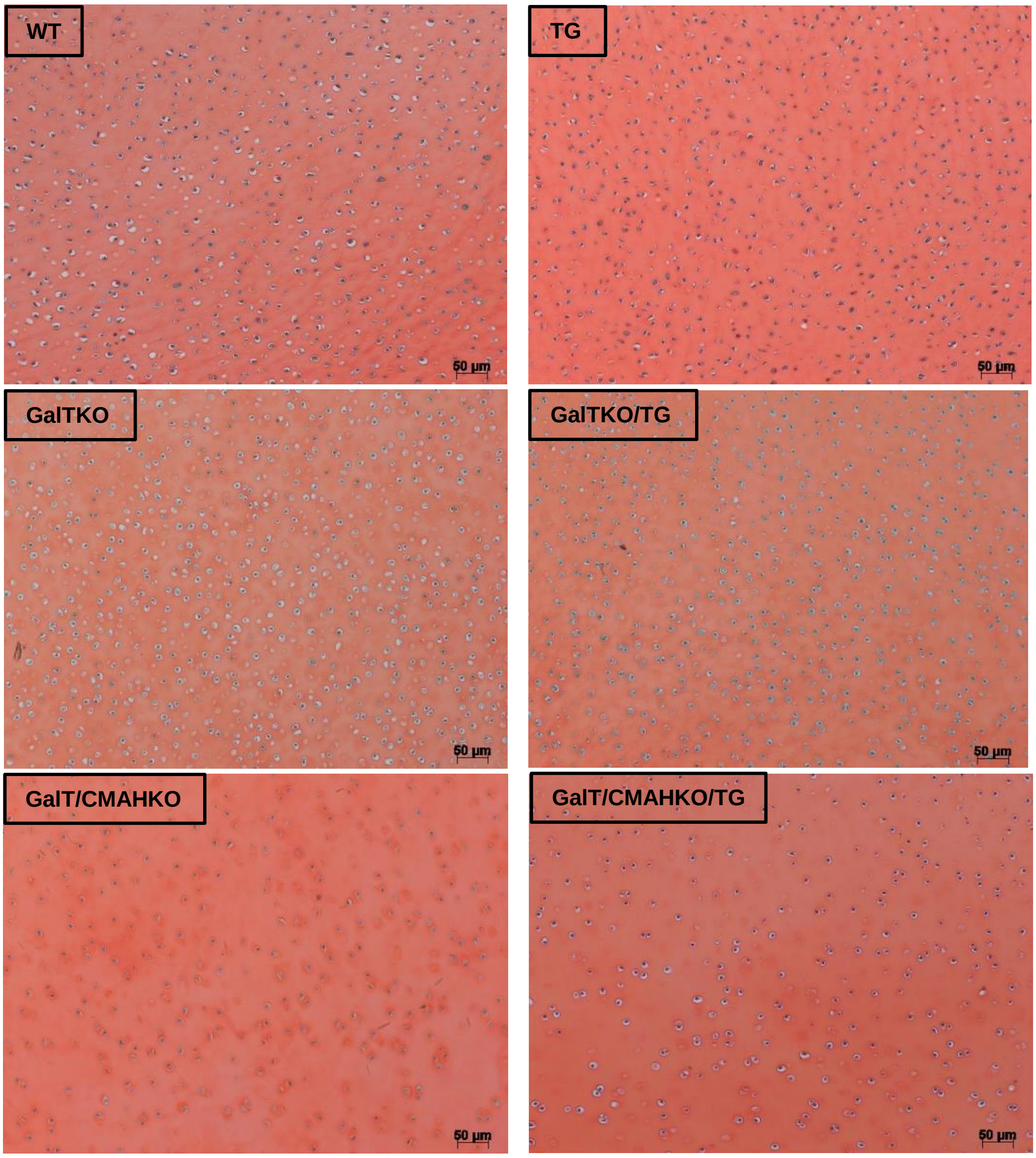

C) Collagen type II staining

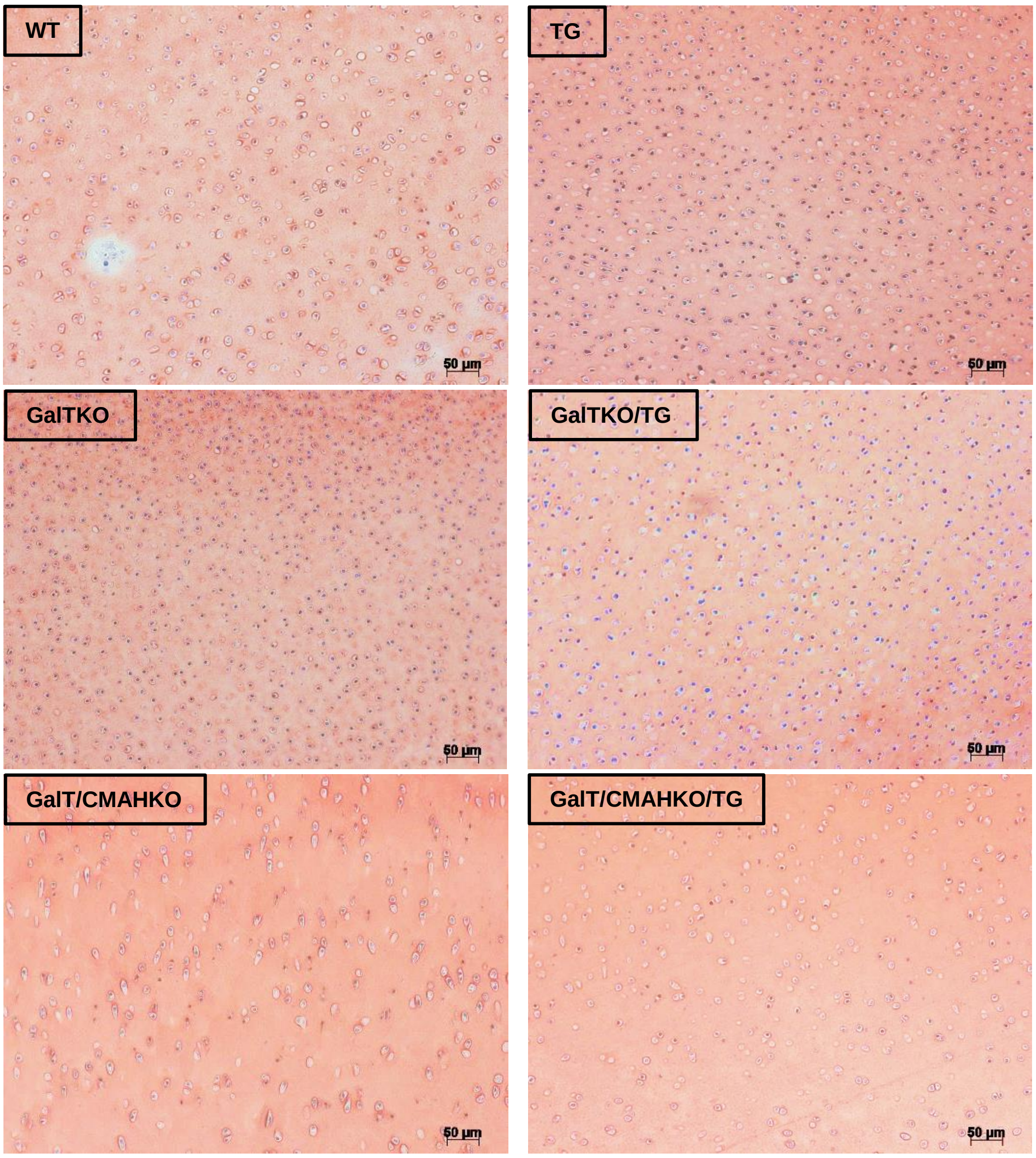

Supplementary S1 Macroscopic and histomorphologic analysis of distal femoral condyles. Evaluation of cartilage appearance (A), as well as proteoglycan visualization with Safranin O staining (B; red coloured) and immunohistological staining of collagen type II (C; red colored, counterstaining with Meyer´s hemalum).

Supplementary Figure S2

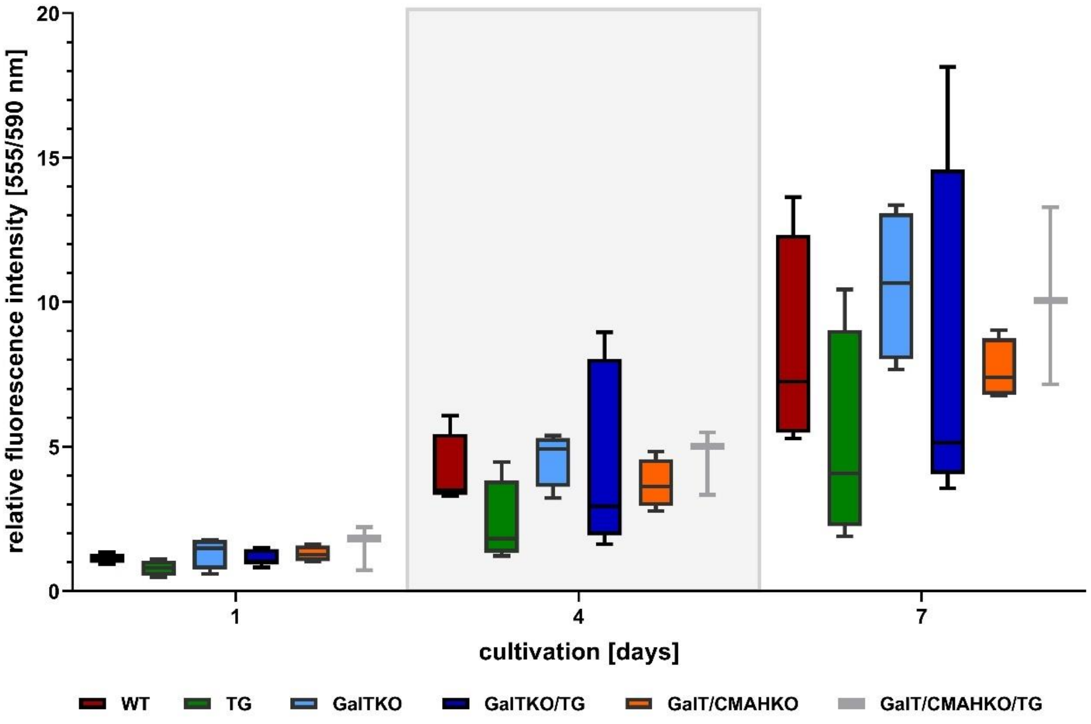

Supplementary S2 Proliferation of multi-genetically modified chondrocytes. Cell growth was evaluated within one week cultivation using alamarBlue assay. Proliferation was determined at culture day 1, 4 and 7 and results are given in relation to t0. No statistical differences compared to WT could be detected by two-way ANOVA with Dunnett´s multiple comparisons test, n=3-5.

Supplementary Figure S3

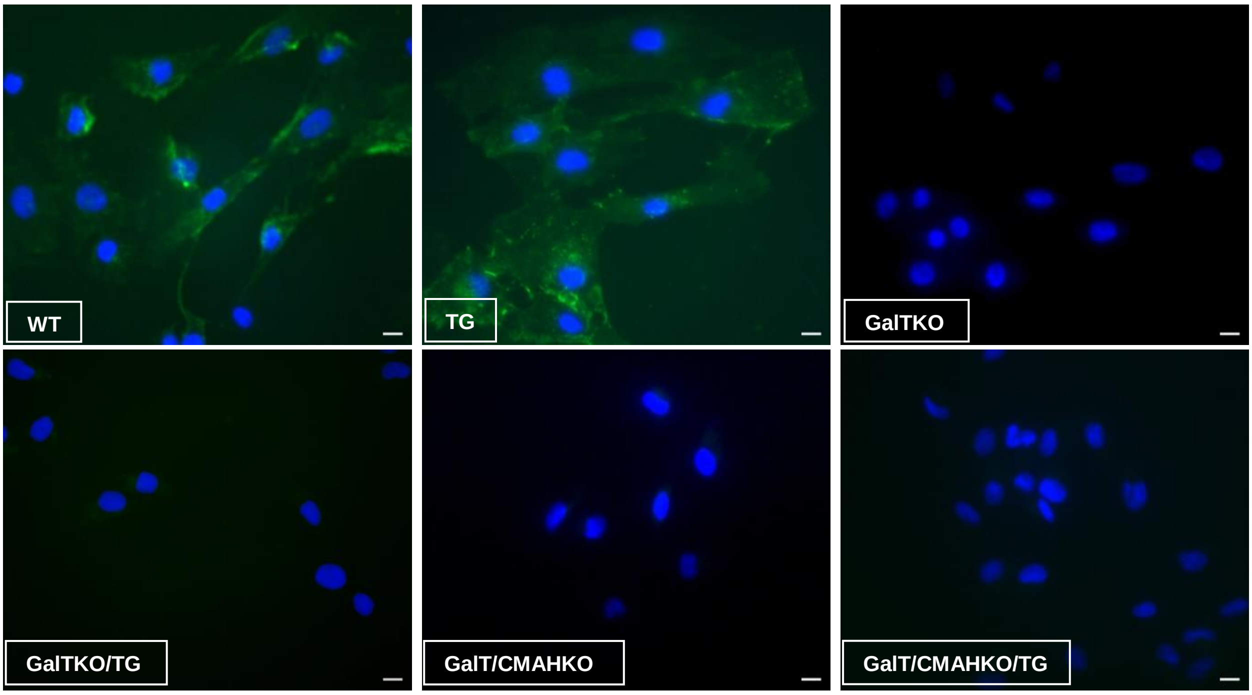

Supplementary S3 Distribution of α-1,3-Gal xenoantigen on porcine chondrocytes. Isolectin B4 FITC conjugated was used to visualize the presence of α-Gal epitope on the cell surface by fluorescence staining (green). Cell nuclei were counterstained with DAPI (blue). Scale bars in the images represent 10 µm.

Supplementary Figure S4

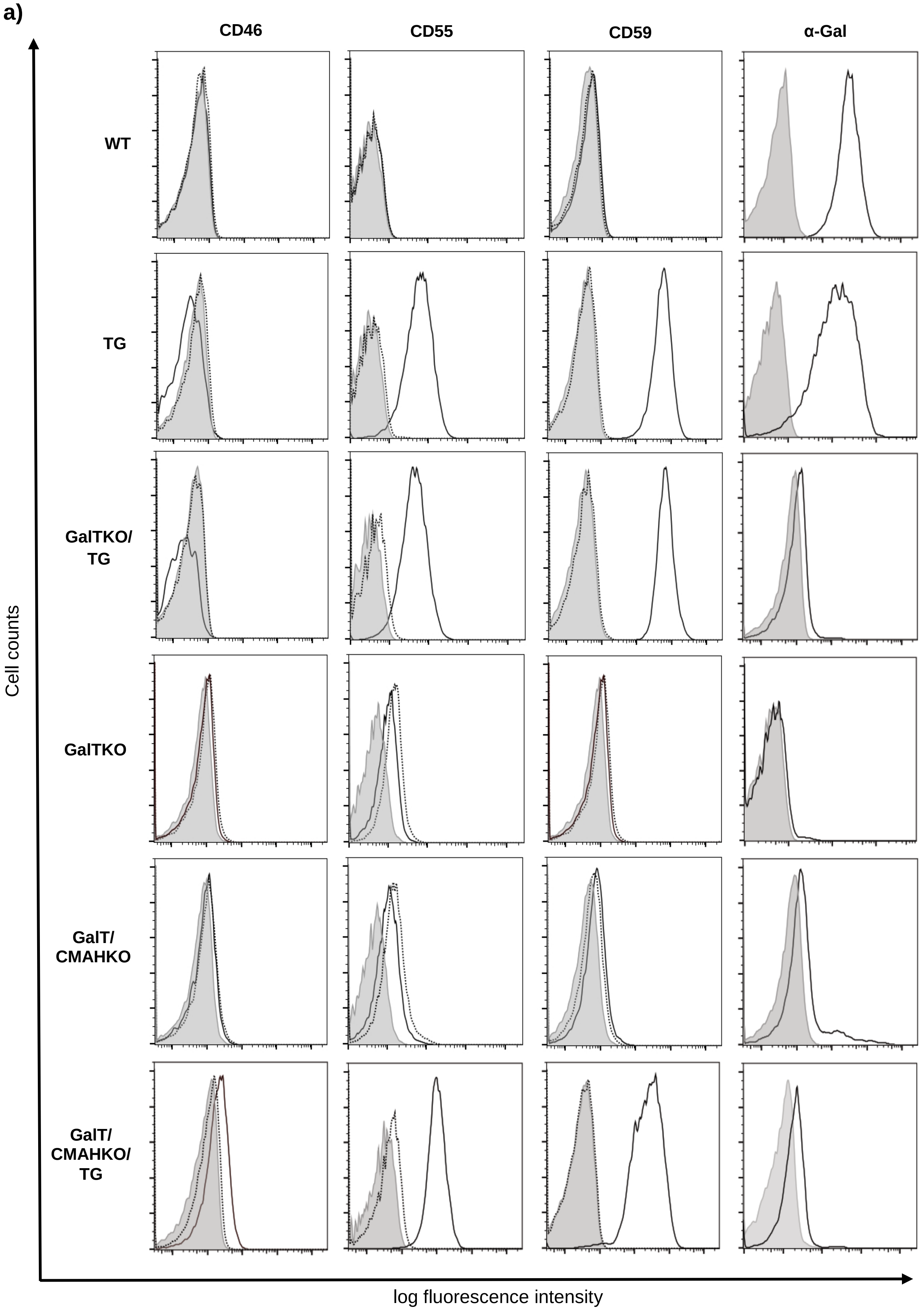

b)

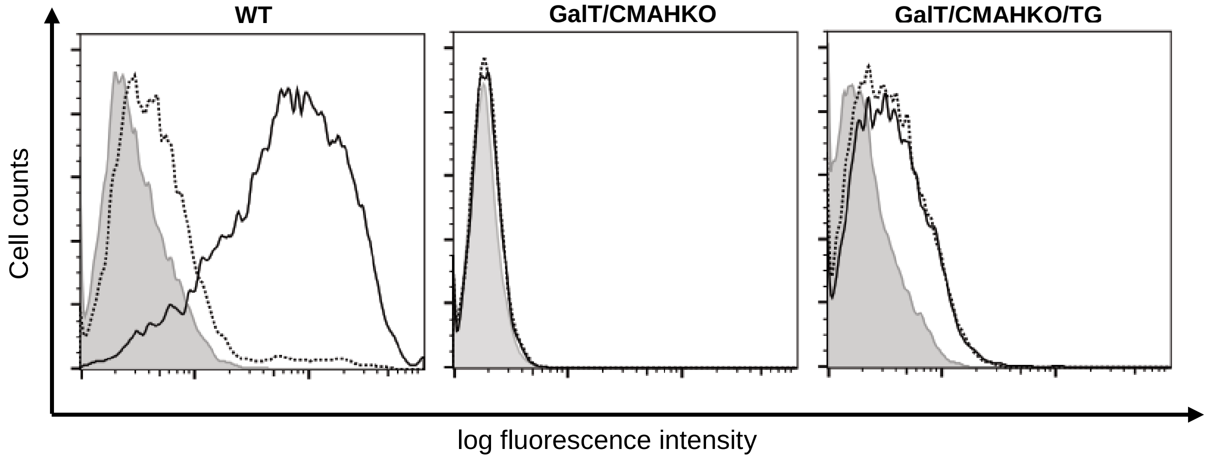

Supplementary S4 Representative histograms of transgene, α-1,3-Gal and Neu5Gc flow cytometric analysis. a) Surface expression of hCregs (CD46, CD55, CD59), α-1,3-Gal and b) Neu5Gc (solid line, respectively). Grey histograms indicate unstained cells and staining with isotype controls are depicted as dotted histograms. 10.000-15.000 cells were measured.

Supplementary Figure S5

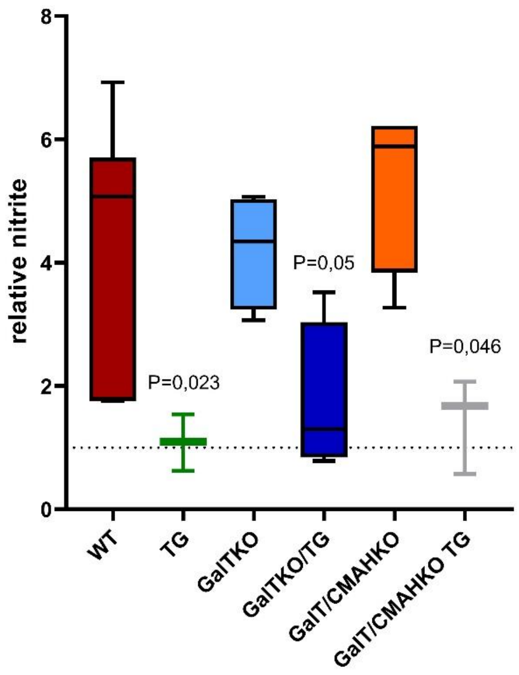

Supplementary S5 Indirect human TNFAIP3 verification in genetically modified porcine chondrocytes via TNF treatment. Porcine chondrocytes were stimulated with 5 ng/ml TNF for 24 h and collected supernatants were analyzed for their nitrite content by Griess Assay. Nitrite concentration was normalized to DNA-content and the results are given in relation to unstimulated cells (dotted line). Statistical analysis was done by two-way ANOVA with Dunnett´s multiple comparisons test. P-values are shown in case of significant differences compared to WT, n=3-5.

Supplementary Table S1

Table S1: Oligonucleotide primers for RT-PCR

a) Species: Homo sapiens (sequences according to Fischer et al. [15])

Gene	Forward/ Reverse	Sequence 5`-3`	Amplicon size (bp)
CD46	F	CAA GCA GTC CCT GCA AAT GG	335
	R	GCA GCA CGA CTC CAC ACT GA	
CD55	F	TTG TCC AGC ACC ACA AA	315
	R	CGT GTT GCT TGA GCA TTT GG	
CD59	F	GGA GTT GAG ACC TAC TTC ACA G	208
	R	CTT TGG TAA TGA GAC ACG CAT	
HMOX1	F	GAT GGA GCG TCC GCA ACC	349
	R	CTT CAC ATA GCG CTG CAT GGC	
TNFAIP3	F	TGG GAC TCC AGA AAA CAA GG	231
	R	GTC CTT TTG GCC TCA TGA AA	

b) Species: Sus scrofa (sequences according to Sommaggio et al. [50])

Gene	Forward/ Reverse	Sequence 5`-3`	Amplicon size (bp)
ACAN	F	CCC ACT AGT GCA GCA ACA GA	191
	R	AGG GTA GAT GGC TGC TCT GA	
COL2A1	F	CAG GGG TGA ACG AGG TTT C	190
	R	AAT ACC AGC AGC TCC CCT CT	
GAPDH	F	GGC GTG AAC CAT GAG AAG TAT G	60
	R	GGT GCA GGA GGC ATT GCT	

Supplementary Figure S6

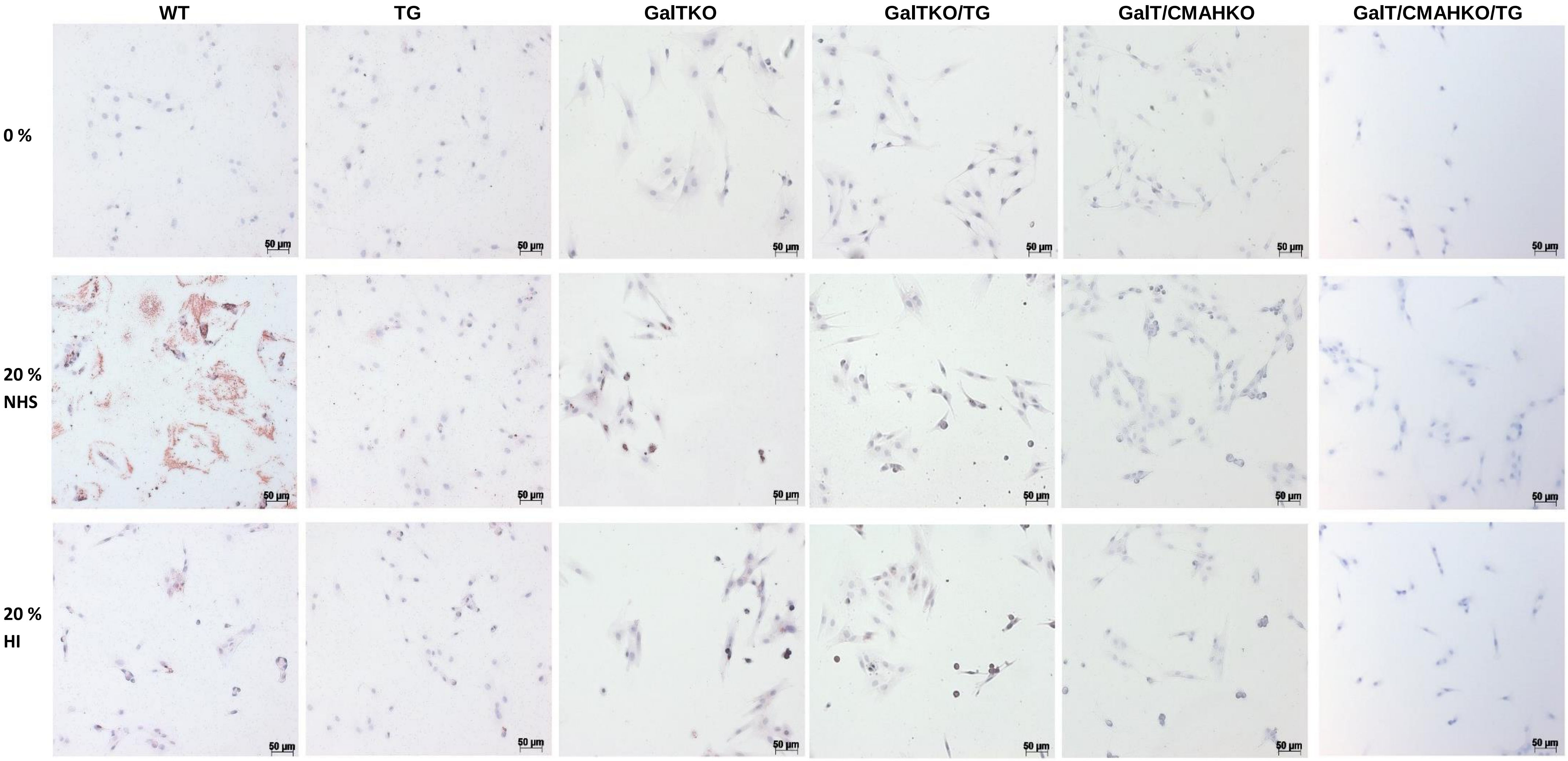

Supplementary S6 C5b-9 deposition on porcine chondrocytes after NHS treatment. Porcine Chondrocytes of all variants were exposed to 20 % NHS w/o heat-inactivation for 1 h. Afterwards immune-cytological staining of membrane-bound C5b-9 was performed (red colored). Meyer´s hemalum was used for counterstaining. Abbreviations: NHS=normal human serum, HI=heat-inactivated NHS.
